# The human T-cell leukemia virus type I (HTLV-I) oncoprotein Tax promotes persistent NF-κB activation by blocking the phosphorylation of the adaptor molecule TAX1BP

**DOI:** 10.1186/1742-4690-8-S1-A158

**Published:** 2011-06-06

**Authors:** Noula Shembade, Edward W Harhaj

**Affiliations:** 1 Department of Microbiology and Immunology, University of Miami, Miami, FL, 33136, USA

## 

Persistent activation of the transcription factor NF-κB by HTLV-1 Tax plays a critical role in T-cell transformation and the onset of adult T-cell leukemia (ATL). Tax activates NF-κB by binding to the NEMO/IKKgamma subunit of the IKK complex, thus leading to persistent IKK activation and nuclear translocation of NF-κB dimers. In addition, Tax counteracts negative regulators of NF-κB such as A20 although the underlying mechanisms remain unclear. We have previously reported that the Tax interacting protein TAX1BP1 serves as a scaffold molecule for the A20 ubiquitin-editing complex, consisting of A20, TAX1BP1, Itch and RNF11, that is critical for the negative feedback of NF-κB. Here we demonstrate that the IKKalpha subunit of IKK phosphorylated TAX1BP1 at Ser593 and Ser624 in response to proinflammatory cytokine stimulation. TAX1BP1 phosphorylation by IKKalpha is essential for the inducible interactions between TAX1BP1, A20, Itch and RNF11 and the subsequent NF-κB inhibitory function of A20. Interestingly, HTLV-I Tax potently blocks TAX1BP1 phosphorylation by preventing the recruitment of IKKalpha to TAX1BP1. Therefore, Tax promotes constitutive NF-κB signaling by inhibiting TAX1BP1 phosphorylation, thus blocking the assembly of the A20 ubiquitin-editing complex and impairing the function of A20.

